# Radiotherapy for Non-Hodgkin’s lymphoma with cardiac infiltration: A case report

**DOI:** 10.3389/fonc.2023.1234831

**Published:** 2023-10-17

**Authors:** Weifeng Wang, Zhuo Zhang, Xiaocong Deng, Anqi Gu, Xianzhao Chen, Yizheng Cai, Yuting Zhao

**Affiliations:** Department of Radiation Oncology, Hainan Cancer Hospital, Haikou, China

**Keywords:** Non-Hodgkin’s lymphoma, cardiac infiltration, radiotherapy, zanubrutinib, case report

## Abstract

**Background:**

Non-Hodgkin’s lymphoma (NHL) with cardiac infiltration has a poor prognosis. The median OS of patients failing to respond to chemotherapy has been reported to be 1 month vs. 18 months in patients responding to chemotherapy.

**Case presentation:**

Herein, we reported a case of a 57-year-old male confirmed with diffuse large B-cell lymphoma who received radiation therapy of 150-cGy daily, administered in 30 fractions to the volume of cardiac infiltration, resulting in complete relief. Chemotherapy had no curative effect. The patient was subsequently enrolled in a clinical trial and received oral administration of zanubrutinib 80mg twice daily, after which he achieved complete remission. The progression-free survival was from diagnosis (January 7, 2020) to the follow-up (September 20, 2022), amounting to 32 months.

**Conclusion:**

Proper irradiation dose and timing of treatment can relieve NHL symptoms.

## Introduction

Non-Hodgkin’s Lymphoma (NHL) is a group of lymphoproliferative disorders that arise from the primary lymphoid system and are characterized by abnormal clonal proliferation of B lymphocytes, T lymphocytes, or Natural Killer (NK) cells (1). Primary extranodal NHL, which affects organs such as the spleen and liver, is rather prevalent in China. When extranodal organ invasion occurs in NHL, the prognosis is usually poor. NHL with cardiac infiltration is rare, and cardiac involvement in malignant NHL is even rarer. In fact, at autopsy, it is found in only about 10% of cases (2).

Herein, we presented a case report of a 57-year-old male patient with refractory NHL. Infiltration of lymphoma cells in the myocardium caused critical conditions with cardiac tamponade, where chemotherapy was ineffective. After receiving radiotherapy and symptomatic supportive treatment, the patient experienced remission and overcame the life-threatening condition. The patient gained an opportunity for further targeted therapy for lymphoma, achieving an eventual progression-free survival of 32 months.

Our report highlights the importance of an aggressive and individualized treatment approach for NHL patients with cardiac invasion, which can lead to satisfactory results. Accumulating diagnosis and treatment experience is essential to improve the understanding of this rare condition.

## Case presentation

Herein, we introduced a case of a 57-year-old male who initially presented for lumbar pain accompanied by anal and urethral bloating pain lasting for two weeks. Later, the patient developed perineal and anal pain, with symptoms worsening over time. His lower limb activity and bowel and urine function were unaffected upon admission. The patient had tenderness in the lumbosacral area but no abnormalities during anal digital examination. He had no prior similar symptoms, medical history of cardiovascular or urinary diseases, or previous treatments.

On January 7, 2020, computed tomography (CT) scan of the chest revealed a mediastinal mass and multiple mediastinal and supraclavicular lymph nodes of varying sizes, with a maximum diameter of approximately 3.0 x 2.5 cm. Lumbar magnetic resonance imaging (MRI) showed multiple space-occupying lesions near the left lumbar major muscle, the L4 vertebra, the abdominal aorta, and the left iliac vessel. Positron emission tomography-CT (PET/CT) confirmed that multiple lymph node regions were invaded, and the fourth lumbar spine and adnexa were invaded by the tumor and compressed the cauda equina nerve. Laboratory examination showed a high level of LDH at 470 U/L (normal range: 104-226 U/L).

**Figure 1:**
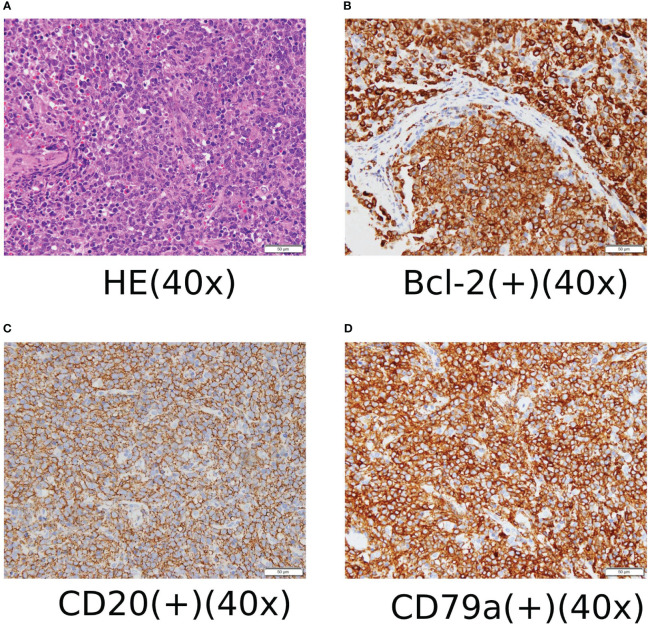
**(A)** HE staining and immunohistochemical staining ×400. **(B)** Some cells expressed Bcl-2 positive immunohistochemical staining ×400. **(C)** Most cells expressed CD20-positive immunohistochemical staining ×400. **(D)** Most cells expressed CD79a positive immunohistochemical staining ×400.

On January 19, 2020, the patient underwent retroperitoneal lymph node dissection and biopsy, which revealed diffuse large B-cell lymphoma of the left iliac perivascular lymph node. Immunohistochemical analysis showed CD3 (–), CD20(+), CD5(+), CD79a (+), CD10 (–), Ki-67 (90%), Mum-1(+),Bcl-2(+),Bcl-6(+),CyclinD1(-), CD30(-), ALK(1A4) (-), CD21(FDC-), and c-MYC (10%+) (see **Figure 1**). FISH results suggested that Bcl-2 (18q21), c-MYC (8q24) chromosomal translocation, and Bcl-6 (3q27) gene rearrangements occurred.

## Treatment

Based on the patient’s medical history and data, the clinical diagnosis and staging were diffuse large B-cell NHL (stage IV-E). In order to relieve pain symptoms, the patient began receiving radiation therapy on January 25, 2020, which consisted of one daily 300-cGy dose for 10 fractions using intensity-modulated radiotherapy to tumors with L4 vertebral and intraspinal metastases. One week after the radiotherapy, the patient received the R-CHOP regimen (intravenously rituximab 375mg/m^2^, cyclophosphamide 750mg/m^2^, doxorubicin 40mg/m^2,^ and vincristine 1.4mg/m^2^ on day 1, and prednisone 100mg/m^2^ from day 1 to 5) from February 12, 2020, to July 20, 2020, for up to 8 cycles. During chemotherapy, the patient developed grade III myelosuppression but recovered after treatment with G-CSF-stimulated white blood cells and successfully completed the entire chemotherapy schedule. As indicated by PET/CT, the tumor underwent partial remission, assessed according to RECIST, version 1.1. Upper Mediastinal and supraclavicular fossa lymph nodes showed varying degrees of reduction, and the largest diameter of lymph nodes was reduced by > 50%. Radiotherapy to the involved field of upper mediastinal and supraclavicular residual lymph nodes and para-aortic lymph nodes were designed as 2Gy per fraction with five daily fractions per week for 5 weeks. After radiotherapy, the tumors showed a complete response.

**Figure 2:**
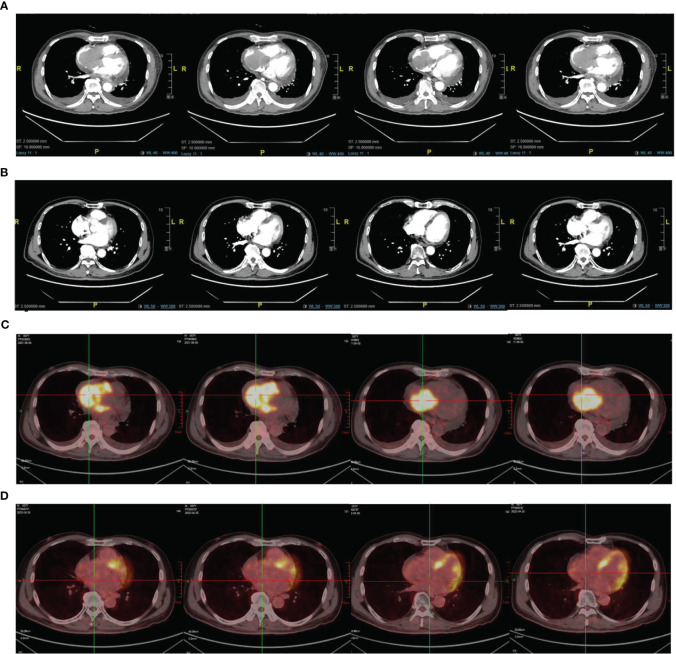
**(A)** Image of chest CT scan on August 3, 2021. **(C)** PET/CT scan on August 6, 2021. **(B)** Image of chest CT scan on April 3, 2022 **(D)** PET/CT scan on April 6, 2022.

Eight months after the radiotherapy, the patient was readmitted for “facial swelling and shortness of breath,” which was caused by tumor invasion of the right atrial tamponade with diameter of approximately 3.0 x 4.5 cm and was confirmed by cardiac ultrasonography and chest enhanced-CT scan (see **Figure 2A**).

Echocardiography showed that the tumor had invaded the right atrium and ventricle, and the tricuspid valve was not involved and the right ventricular FAC < 35% and Tei index > 0.55. Right ventricular function decreased significantly. PET/CT confirmed multiple areas of abnormal radiation concentration in the mediastinum, right atrium, and right ventricle (SUVmax = 16.2). It was considered that the progressive tumor invasion of the right atrium and right ventricle caused the patient’s symptoms (see **Figures 2A, C**).

After one cycle of CHOP regimen (cyclophosphamide 750mg/m2, doxorubicin 40mg/m2, vincristine 1.4mg/m2, and prednisone 100mg/m2, D1-5), the patient’s symptoms worsened, including facial edema, chest tightness, and shortness of breath. Following chemotherapy, the patient’s symptoms aggravated for the following reasons: the patient had symptoms of cardiac tamponade, which was aggravated after the intravenous infusion. In addition, the patient developed chemotherapy resistance after a long course of CHOP chemotherapy. After evaluating his cardiac, pulmonary, and coagulation functions, cardiac myxoma was excluded, and radiotherapy was used to alleviate the symptoms. Radiotherapy was initiated on August 13, 2021, targeting the right atrium, right ventricle, and lower mediastinal lymph nodes. Gross tumor volume (GTV), which is defined as a tumor with a precise location and range, making the tumor visible in imaging scans, included lower mediastinal lymph nodes, tumors in the right atrium and right ventricle, and tumors around the superior vena cava. The PTV was externally extended by 0.5cm in the front, rear, left, and right, and 0.8cm in the top and bottom of GTV. Palliative radiotherapy was given, and no CTV was set.

As the heart is a late-response tissue sensitive to fractionated doses, a single 1.5Gy dose was administered in 30 fractions (DT=45Gy) to minimize radiation-related damage. This radiotherapy and mediastinal radiation were used on previous plans to integrate and assess the radiotherapy risk of the heart, spinal cord, trachea, esophagus, and other at-risk organs at tolerable doses. The prescription dose was GTV: 45Gy/30F, with Heart V20 = 50.6%, V30 = 40.3%, Dmax=4524cGy, and Dmean=1613cGy, Spinal cord Dmax= 3021cGy Trachea Dmax=4438cGy, Esophagus Dmax=4462cGy. During radiotherapy, the patient was given oral rivaroxaban 20mg daily for three months to prevent pulmonary embolism caused by tumor necrosis and tumor thrombus. After completing Dt=21.6Gy/12F, the localization was re-simulated. The target volume was re-delineated based on the tumor regression, and a new radiotherapy plan was made. Symptomatic and supportive treatments were provided during treatment.

## Follow-up and outcome

After radiotherapy, echocardiography confirmed that the atrial and intraventricular tumors had regressed entirely. However, one month later, several enlarged lymph nodes were found in the right supraclavicular area, with the largest size of about 3*2.5 cm. Due to disease progression, chemotherapy was no longer an option. A needle rebiopsy of the lymph node was performed, and gene detection confirmed CD79B (Y196 mut) mutation. The patient was enrolled in the clinical trial for the treatment of CD79B Mutant Relapsed/Refractory Diffuse Large B-Cell lymphoma with Bruton Tyrosine Kinase Inhibitor Zanubrutinib (Clinical Trial NO: NCT 05068440). The patient was given oral zebrutinib (80mg twice daily) on October 8, 2021, and regular reexamination showed that the tumor gradually regressed. On April 24, 2022, PET/CT and chest CT revealed that the tumor was in complete remission (see **Figures 2B, D**). The patient is currently receiving oral zebrutinib treatment and is under regular follow up. The last follow-up was on November 20, 2022, and the response was evaluated as a stable disease.

## Discussion

NHL is a common malignant tumor that originates from lymph nodes and lymphoid tissues. The main factors affecting poor prognosis are poor general condition, mass > 10cm, extranodal invasion of more than 2 organs, fever and night sweats, weight loss, elevated serum lactate dehydrogenase, and advanced age (3).

Lymphoma in the heart includes primary cardiac lymphoma and cardiac lymphoma invasion. The incidence of NHL is about 20% (4), accounting for 2% of cardiac malignant tumors (5). Cardiac involvement by malignant NHL (primary lymphoma of the heart) is extremely rare. Cardiac lesions occurring during malignant NHL (secondary lymphoma of the heart) are found at autopsy in approximately 10% of cases (6). Among 94 patients with biopsy-proven NHL with cardiac involvement (7), diffuse large B-cell lymphoma (DLBCL) was found to be the most common (58%), followed by T-cell lymphoma (16%) and Burkitt lymphoma (9%). Cardiac secondary lymphoma may involve all cardiac structures, with lesions in the right atrium being dominant. The clinical presentation is usually nonspecific, with diffuse involvement occurring in advanced stages (8).

Cardiac lymphoma can cause various clinical manifestations depending on multiple factors, including the location, size, growth rate, degree of invasion, and mass fragility of the tumor (7). Intracardiac masses can obstruct blood flow, cause tumor embolism, or affect valve function. If the tumor invades the pathway, it can cause arrhythmia. If the pericardium is involved, it can lead to pericardial effusion or tamponade. Involvement of the myocardium can cause an increase in myocardial enzymes. A retrospective study found that about 34% of patients with lymphoma invading the heart developed heart failure, while 20% had no cardiovascular-related symptoms. Another study (7) showed that patients who did not receive chemotherapy had a median survival time of only 1 month vs. 18 months in those who did.

In this case, the patient had lymphoma invading the right atrium and right ventricle, which significantly decreased the pumping function of the heart and cardiovascular system-related symptoms. PET-CT showed that the tumor did not cause diffuse cardiac invasion, but there were apparent space-occupying lesions in the cardiac cavity. One cycle of chemotherapy was ineffective, so local palliative radiotherapy was used. After evaluation of the heart, lung function, bleeding, and coagulation function, and excluding cardiac myxoma, palliative radiotherapy was used to treat cardiac involvement. Since the heart is sensitive to fractionated doses, a single fraction of 1.5Gy was used in 30 fractions (DT=45Gy) to reduce late radiation-induced toxicity. The dose of cardiac radiation was strictly limited during radiotherapy, and the tumor regression of cardiac involvement was dynamically detected. The target volume was timely relocated to reduce the irradiation target volume and the dose to the heart. Anticoagulant drugs were orally taken to prevent tumor cell necrosis and embolism. No severe complications occurred during the entire treatment.

Based on the treatment process and follow-up results of this case, we can conclude that radiotherapy is still the preferred local treatment after the failure of chemotherapy for radiation-sensitive tumors. Even if the tumors invade critical organs, active and prudent radiotherapy can still obtain satisfactory results, quickly relieve the disease, and provide patients with subsequent treatment opportunities. In cases where critical organs poorly respond to radiotherapy, the radiotherapy method and plan should be rationally arranged according to the radiobiological characteristics of organs and tissues. Adequate evaluation and intervention with necessary anticoagulant drugs can minimize the risk of thrombosis, thereby avoiding fatal complications. After the regression of the tumor involving the heart, the patient’s cardiac function returned to normal, and he was successfully enrolled in a clinical trial. The patient responded well to zebrutinib treatment, and the tumors involving other lymphatic regions have regressed satisfactorily.

## Conclusion

Compared to patients with NHL with cardiac infiltration in previous literature, our patient achieved long-term survival, thus representing a unique case. Proper irradiation dose and timing of treatment can relieve symptoms and provide opportunities for follow-up treatment of patients with NHL invading the heart. Early diagnosis and treatment are critical for long-term survival and improved quality of life.

## Data availability statement

The original contributions presented in the study are included in the article/**Supplementary Material**. Further inquiries can be directed to the corresponding author.

## Ethics statement

Ethical approval was not required for the studies involving humans because Since this study was retrospective, ethical approval was waived. The studies were conducted in accordance with the local legislation and institutional requirements. The participants provided their written informed consent to participate in this study. Written informed consent was obtained from the individual(s) for the publication of any potentially identifiable images or data included in this article.

## Author contributions

WW and ZZ carried out the studies, participated in collecting data, and drafted the manuscript. AG and XC performed the statistical analysis and participated in its design. YC and YZ participated in acquisition, analysis, or interpretation of data and draft the manuscript. All authors contributed to the article and approved the submitted version.
