# Supplementary material for: Radiotherapy for Non-Hodgkin’s lymphoma with cardiac infiltration: A case report
Source: Front Oncol. 2023 Oct 17;13:1234831. doi: 10.3389/fonc.2023.1234831 (PMC10616872; doi:10.3389/fonc.2023.1234831)
Supplement: Supplementary file 1 [file Table_1.docx]

| **Topic** | **Item** | **Checklist item description** | **Reported on page** |
| --- | --- | --- | --- |
| **Title** | **1** | The words “case report” should be in the title along with what is of greatest interest in this case | Page-1 |
| **Key Words** | **2** | The key elements of this case in 2 to 5 key words | Page-1 |
| **Abstract** | **3a** | Introduction—What is unique about this case? What does it add to the medical literature? | Page-1 |
|  | **3b** | The main symptoms of the patient and the important clinical findings | Page-1 |
|  | **3c** | The main diagnoses, therapeutics interventions, and outcomes | Page-1 |
|  | **3d** | Conclusion—What are the main “take-away” lessons from this case? | Page-1 |
| **Introduction** | **4** | Brief background summary of this case referencing the relevant medical literature | Page-2 |
| **Patient Information** | **5a** | Demographic information (such as age, gender, ethnicity, occupation) | Page-2 |
|  | **5b** | Main symptoms of the patient (his or her chief complaints) | Page-2 |
|  | **5c** | Medical, family, and psychosocial history including co-morbidities, and relevant genetic information | Page-2 |
|  | **5d** | Relevant past interventions and their outcomes | Page-2 |
| **Clinical Findings** | **6** | Describe the relevant physical examination (PE) findings | Page-2 |
| **Timeline** | **7** | Depict important milestones related to your diagnoses and interventions (table or figure) | Page-2 |
| **Diagnostic Assessment** | **8a** | Diagnostic methods (such as PE, laboratory testing, imaging, questionnaires) | Page-2 |
|  | **8b** | Diagnostic challenges (such as financial, language, or cultural) |  |
|  | **8c** | Diagnostic reasoning including other diagnoses considered |  |
|  | **8d** | Prognostic characteristics (such as staging in oncology) where applicable | Page-2 |
| **Therapeutic Intervention** | **9a** | Types of intervention (such as pharmacologic, surgical, preventive, self-care) | Page-2 |
|  | **9b** | Administration of intervention (such as dosage, strength, duration) | Page-2 |
|  | **9c** | Changes in intervention (with rationale) |  |
| **Follow-up and Outcomes** | **10a** | Clinician-assessed outcomes and when appropriate patient-assessed outcomes | Page-3 |
|  | **10b** | Important follow-up test results | Page-3 |
|  | **10c** | Intervention adherence and tolerability (How was this assessed?) | Page-3 |
|  | **10d** | Adverse and unanticipated events |  |
| **Discussion** | **11a** | Discussion of the strengths and limitations in the management of this case | Page-3 |
|  | **11b** | Discussion of the relevant medical literature | Page-3 |
|  | **11c** | The rationale for conclusions (including assessment of possible causes) | Page-4 |
|  | **11d** | The main “take-away” lessons of this case report |  |
| **Patient Perspective** | **12** | Did the patient share his or her perspective or experience? (Include when appropriate) |  |
| **Informed Consent** | **13** | Did the patient give informed consent? Please provide if requested | **Yes _√_ No ___** |

**CARE Checklist (2013) of information to include when writing a case report**
